# Persistence of analgesic usage and opioid consumption in sarcopenic patients undergoing neuraxial anesthesia: a nationwide retrospective cohort study

**DOI:** 10.1097/PR9.0000000000001129

**Published:** 2024-03-08

**Authors:** Yitian Yang, Wan-Ming Chen, Szu-Yuan Wu, Jiaqiang Zhang

**Affiliations:** a Department of Anesthesiology and Perioperative Medicine, Henan Provincial People's Hospital, People's Hospital of Zhengzhou University, Zhengzhou, China; b Graduate Institute of Business Administration, College of Management, Fu Jen Catholic University, Taipei, Taiwan; c Artificial Intelligence Development Center, Fu Jen Catholic University, Taipei, Taiwan; d Department of Food Nutrition and Health Biotechnology, College of Medical and Health Science, Asia University, Taichung, Taiwan; e Big Data Center, Lo-Hsu Medical Foundation, Lotung Poh-Ai Hospital, Yilan, Taiwan; f Division of Radiation Oncology, Lo-Hsu Medical Foundation, Lotung Poh-Ai Hospital, Yilan, Taiwan; g Department of Healthcare Administration, College of Medical and Health Science, Asia University, Taichung, Taiwan; h Cancer Center, Lo-Hsu Medical Foundation, Lotung Poh-Ai Hospital, Yilan, Taiwan; i Centers for Regional Anesthesia and Pain Medicine, Taipei Municipal Wan Fang Hospital, Taipei Medical University, Taipei, Taiwan; j Department of Management, College of Management, Fo Guang University, Yilan, Taiwan

**Keywords:** Chronic postsurgical pain, Long-term postsurgical analgesic use, Neuraxial anesthesia, Sarcopenia, Nonsarcopenia

## Abstract

**Introduction:**

This study investigates the association between chronic postsurgical pain (CPSP) and long-term postsurgical analgesic usage in patients undergoing neuraxial anesthesia, with a specific focus on the presence or absence of sarcopenia.

**Objectives:**

To assess the rate of analgesic prescription, including opioids, at 3 and 6 months postsurgery for patients with and without preoperative sarcopenia, and to determine the impact of sarcopenia on analgesic use after neuraxial anesthesia surgery.

**Methods:**

Patients undergoing surgery under neuraxial anesthesia were categorized into sarcopenic and nonsarcopenic groups based on preoperative diagnosis using the ICD-10-CM code M62.84. Propensity score matching in a 1:4 ratio was applied for group matching. Analgesic prescription rates were evaluated at 3 and 6 months postsurgery, and multivariable logistic regression was used to analyze analgesic use, comparing patients with and without preoperative sarcopenia.

**Results:**

Among 3805 surgical patients, 761 had sarcopenia, while 3044 did not. At 3 months postsurgery, 62.3% of sarcopenic patients received analgesics, with 2.9% receiving opioids, compared to 57.1% of nonsarcopenic patients receiving analgesics and 0.8% receiving opioids. At 6 months postsurgery, 30.8% of sarcopenic patients received analgesics (1.7% opioids), while 26.3% of non-sarcopenic patients received analgesics (0.3% opioids). Multivariable logistic regression analysis revealed that preoperative sarcopenia was significantly associated with higher analgesic prescription rates at both 3 months (adjusted odds ratio [aOR], 1.27; 95% confidence interval [CI], 1.05–1.53) and 6 months (aOR, 1.17; 95% CI, 1.07–1.42) postsurgery. Furthermore, sarcopenic patients exhibited significantly higher opioid prescription rates at 3 months (aOR, 1.11; 95% CI, 1.05–2.45) and 6 months (aOR, 1.89; 95% CI, 1.12–4.96) postsurgery.

**Conclusion:**

Sarcopenia emerges as an independent risk factor for prolonged analgesic use after neuraxial anesthesia surgery and significantly elevates the risk of developing CPSP.

## Abstract 2

Supplemental Digital Content is Available in the Text.

Sarcopenia in patients undergoing surgery under neuraxial anesthesia increases the risk of chronic postsurgical pain and long-term analgesic use.

## 1. Introduction

Sarcopenia is characterized by the progressive loss of skeletal muscle mass and strength, which can have a negative impact on patients' quality of life, physical function, and overall health.^8^ The underlying causes of sarcopenia remain unclear, but contributing risk factors include unhealthy lifestyle habits, malnutrition, physical inactivity, and chronic illness.^7^ Sarcopenia can also affect the occurrence, progression, and prognosis of various diseases and increase the risk of falls, disability, hospitalization, and mortality.^21^ In addition, patients with sarcopenia are often older adults with limited physical function, which can increase their susceptibility to postoperative complications.^9^ Given these risks, it is crucial for surgeons and anesthesiologists to closely monitor and manage patients with sarcopenia during and after surgery to mitigate adverse outcomes.

Postsurgical pain is among the commonest adverse events after surgery. Chronic postsurgical pain (CPSP), which is triggered by surgery and lasts for more than 3 months, may occur if acute postoperative pain is not completely relieved.^15^ The pathogenesis of CPSP remains unclear. In addition to causing physical pain, CPSP can lead to multiple comorbidities, such as sleep disorders, pneumonia, immunosuppression, anxiety, and depression.^19^ Analgesics are often the first choice of treatment for CPSP. However, the long-term use of analgesics or opioids can cause stomach ulcers, liver damage, blood coagulation abnormalities, drug addiction, and cancer.^16,20,24^ Therefore, the prevention of CPSP and reduction of the long-term use of postoperative analgesic drugs are crucial goals.

The correlation between sarcopenia and long-term analgesic and opioid use after surgery under neuraxial anesthesia remains not fully understood, and studies with adequate sample sizes and clear definitions of sarcopenia are limited.^18^ Moreover, no study has investigated the incidence of CPSP and long-term analgesic use in patients with sarcopenia after surgery. Understanding the relationship between sarcopenia and long-term analgesic use is essential, particularly in the context of older patients, who are more vulnerable to sarcopenia. This knowledge is pivotal for tailoring effective pain management strategies while minimizing the potential risks associated with prolonged analgesic, including opioids, use. By investigating this connection, health care providers can achieve a delicate balance between pain relief, improved patient quality of life, and optimized resource allocation. Moreover, given the potential variations in long-term analgesic use between neuraxial and general anesthesia,^32^ a comprehensive study using real-world data is imperative to explore this complex relationship. To bridge this knowledge gap, we conducted a comparative analysis using propensity score matching (PSM) to evaluate the influence of preoperative sarcopenia on long-term analgesic and opioid utilization in patients undergoing surgery under neuraxial anesthesia, ultimately shedding light on its impact on CPSP development.

## 2. Patients and methods

### 2.1. Data source

Data from Taiwan's National Health Insurance Research Database (NHIRD) from January 1, 2016, to December 31, 2019, were used in this study. The follow-up period for patients extended until December 31, 2020. In addition, there was no loss to follow-up within the scope of our study. The Taiwan NHIRD provided comprehensive data until the end of 2020. The NHIRD includes registration and original claims data for all NHI beneficiaries, totaling approximately 27.38 million individuals. To ensure patient privacy, all NHIRD data are encrypted and contain comprehensive outpatient and inpatient claims information, including patient identification numbers, birth dates, sex, diagnostic codes based on the International Classification of Diseases (ICD-9-CM and ICD-10-CM), treatment information, medical costs, dates of hospital admission and discharge, and dates of death.^24,25^ Patient identification numbers were used to link all data sets.

### 2.2. Ethics

Ethical approval for this study (Ethical Committee Tzu-Chi Medical Foundation) was provided by the Ethical Committee Tzu-Chi Medical Foundation, Taiwan (Chairperson Prof Chien-Hsing Wang), on 12 May 2021. The study protocols were reviewed and approved by the Institutional Review Board of Tzu-Chi Medical Foundation (IRB109-015-B). Patient consent was waived because data files are deidentified by scrambling the identification codes of both patients and medical facilities and sent to the National Health Research Institutes to form the original files of NHIRD. This study followed the Strengthening the Reporting of Observational Studies in Epidemiology (STROBE) reporting guideline. This study used retrospective data from Taiwan's NHIRD, a comprehensive source of patient information. Owing to its observational nature and reliance on existing data, preregistration was not pursued.

### 2.3. Design

This retrospective population-based case–control analysis focused on patients who underwent elective surgeries under neuraxial anesthesia between January 1, 2016, and December 31, 2019. This study aimed to explore the potential relationship between sarcopenia and long-term analgesic use after surgery. The surgeries included herniorrhaphy, hip and knee replacements, lower-limb open reduction internal fixation, and lower-limb amputation, chosen for their suitability for neuraxial anesthesia. Patient eligibility for neuraxial anesthesia was determined based on a set of established criteria. These criteria encompassed factors such as the type of surgical procedure, patient suitability, informed consent, thorough preoperative assessments, careful monitoring during surgery, comprehensive documentation, and postoperative care. By adhering to these criteria, health care professionals ensured that neuraxial anesthesia was applied when most appropriate and beneficial, considering both patient safety and surgical efficacy. The anesthesia type was ascertained using the corresponding payment code in Taiwan's NHIRD.^32^ To eliminate the influence of underlying chronic pain and pain from other sources, patients who used analgesics, opioids, or nonopioids for more than 1 month before surgery were excluded. Patients with cancer that may have significantly affected their recovery from surgery or cancer-related pain were also excluded. Patients who passed away within 6 months of surgery were excluded, and the surgeries were performed until December 31, 2019, with follow-up until the end of 2020. The index date was the day of surgery.

In October 2016, the formal recognition of sarcopenia as a disease and its classification as M62.84 in ICD-10-CM was announced by the US Centers for Disease Control and Prevention.^1^ The diagnosis of sarcopenia in our study adhered to the ICD-10-CM code,^25^ which was established after 2016. We used a criterion of at least 2 claims for patients with a principal diagnosis of sarcopenia within the 12-month preoperative period to diagnose sarcopenia.^6^ The coding of sarcopenia in our study was based on a previous research investigation. The definition of sarcopenia was a skeletal muscle mass index (SMI) that was 2 or more standard deviations below the mean values of young individuals of the same sex. Computed tomography images provided the relevant measurements, and the SMI was calculated using the following formula: SMI = L3 skeletal muscle cross-sectional area (cm^2^)/height^2^(m^2^).^30^ Sarcopenia diagnoses made by orthopedic physicians, rehabilitation physicians, family medicine specialists, and geriatricians were included in the sarcopenia group.

The study size in our research was determined based on the retrospective available data within the Taiwan NHIRD over the specified timeframe, in accordance with our inclusion and exclusion criteria. We have included a flow diagram that illustrates the patient selection process, including the number of patients at each stage, which provides clarity regarding our study size determination (Supplemental Figure 1, http://links.lww.com/PR9/A217).

### 2.4. Covariates and propensity score matching

After adjustment for potential confounding factors, a multivariable logistic regression model was used to analyze the use of analgesics 3 or 6 months after surgery under neuraxial anesthesia, comparing patients with and without preoperative sarcopenia. To reduce potential bias when comparing analgesic use between sarcopenia and nonsarcopenia groups, we used PSM to match all patients based on age, sex, surgery type, medical center level, American Society of Anesthesiology (ASA)–derived physical status, coexisting comorbidities, smoking status, alcohol-related diseases (ARDs), Charlson Comorbidity Index (CCI) score, and adapted Diabetes Complications Severity Index (aDCSI) score. We matched the cohorts using a 1:4 ratio and a caliper of 0.1,^2^ and comorbidities were identified using ICD-9-CM and ICD-10-CM codes in the main diagnosis records of inpatients, or with at least 2 outpatient visits within 1 year. Comorbidities that occurred 1 year before the index date were considered. The hospital accreditation level was also taken into account. Medical centers were defined as facilities with 1000 to 2500 beds, providing tertiary medical services and conducting most staff training within the center, as well as having research facilities, in accordance with the Taiwan Joint Commission on Hospital Accreditation.^32^ Owing to the comprehensive nature of the covariates in the Taiwan National Health Insurance Research Database (NHIRD), the occurrence of missing data was minimal, with all covariates containing less than 1% missing data. Consequently, patients with missing data in any of these covariates were excluded from the analysis to ensure data completeness and accuracy.

In our PSM study, we used methods to examine subgroups and interactions, including stratifying the study population into distinct subgroups based on specific covariates of interest for subgroup analysis. Interaction terms were incorporated into our regression models to formally test interactions between treatment variables and key covariates. Sensitivity analyses using different PSM techniques and caliper widths were performed to assess the robustness of our findings.^10^ Forest plots were created to visually present effect sizes and confidence intervals for different subgroups. Formal interaction tests based on interaction terms were conducted to assess the statistical significance of treatment–covariate interactions.^34^

Appropriate continuous variables are presented as means ± standard deviations. To minimize differences among participants, we used a PSM ratio of 1:4 for the preoperative sarcopenia and nonsarcopenia groups, which is commonly used to select controls with identical background covariates. To determine whether preoperative sarcopenia is an independent predictor of high rates of long-term analgesic use in surgical patients, a multivariable logistic regression model was used. Odds ratios (ORs) with 95% confidence intervals (CIs) were calculated by performing the multivariable logistic regression analysis. The analysis was conducted for all analgesics, including opioids.

### 2.5. Outcomes

As CPSP is pain that is specific to surgery and lasts for more than 3 months,^15^ we defined the primary outcome as the combined rate of analgesic prescriptions given 3 and 6 months after surgery. To be classified as a long-term analgesic user, patients needed to receive one or more prescriptions for analgesics, which included acetaminophen, nonsteroidal anti-inflammatory drugs, phenytoin, carbamazepine, pregabalin, gabapentin, and opioids, from outpatient clinics at 3 months or later after surgery. Those who were prescribed opioids such as morphine, fentanyl, oxycodone, buprenorphine, hydromorphone, tramadol, codeine, and meperidine were classified as long-term opioid users.^32^

### 2.6. Statistic

The descriptive findings pertaining to age, sex, surgery type, medical center level, ASA-derived physical status, coexisting comorbidities, smoking status, ARDs, CCI score, and aDCSI score were presented as absolute frequencies with percentages, while standardized differences were used to evaluate the baseline information between the 2 groups. The potential confounders linked to the occurrence of persistent analgesic use were considered as covariates in the predicted model to explore the association between the sarcopenia and nonsarcopenia groups that underwent elective surgery under neuraxial anesthesia. Despite applying PSM using SAS PROC PSM, the residual imbalance might still persist in a population due to a large sample size^33^; hence, we used multivariable logistic regression with SAS PROC LOGISTIC to assess the probability of long-term analgesic use between the groups by estimating adjusted ORs (aORs) and 95% CIs. All statistical analyses were conducted using SAS version 9.4 (SAS Institute Inc, Cary, NC).

## 3. Results

### 3.1. Study cohort

This study analyzed data from 3805 surgical patients undergo neuraxial anesthesia, with 761 in the sarcopenia group and 3044 in the nonsarcopenia group. Table 1 presents their characteristics, including age, sex, surgery type, medical center level, ASA-derived physical status, coexisting comorbidities, smoking status, ARD, CCI score, and aDCSI score. After PSM, there were no significant differences in these characteristics between the groups. The 3-month analgesic and opioid prescription rates were higher in the sarcopenia group compared with the nonsarcopenia group, with rates of 62.3% and 57.1% (*P* = 0.009) and 2.9% and 0.8% (*P* = 0.014), respectively. Similarly, the 6-month analgesic and opioid prescription rates were also higher in the sarcopenia group, with rates of 30.8% and 26.3% (*P* = 0.013) and 1.7% and 0.3% (*P* = 0.030), respectively (Table 1).

**Table 1:** Demographic characteristics of patients receiving elective surgery under neuraxial anesthesia, grouped according to the presence of sarcopenia (after propensity score matching).

	Nonsarcopenia		Sarcopenia		SMD
	N = 3044		N = 761		
	N	%	N	%	
Age					
Age (mean ± SD)	64.14 ± 15.94		64.07 ± 15.86		0.0040
Age, median (IQR), y	67.00 (56.00, 75.00)		67.00 (55.00, 76.00)		
Age group, y					0.0190
<30	131	4.3%	34	4.5%	
30–50	398	13.1%	102	13.4%	
51–70	1,287	42.3%	315	41.4%	
>70	1,228	40.3%	310	40.7%	
Sex					−0.0020
Female	1,586	52.1%	395	51.9%	
Male	1,458	47.9%	366	48.1%	
Surgery type					0.0340
Herniorrhaphy	723	23.8%	182	23.9%	
Hip replacement	130	4.3%	36	4.7%	
Knee replacement	845	27.8%	207	27.2%	
Lower-limb ORIF	1,284	42.2%	318	41.8%	
Lower-limb amputation	62	2.0%	18	2.4%	
Medical center					0.0039
No	2,332	76.6%	586	77.0%	
Yes	712	23.4%	175	23.0%	
ASA-derived physical status					0.0180
1	670	22.0%	167	21.9%	
2	435	14.3%	107	14.1%	
3	1,525	50.1%	379	49.8%	
4	414	13.6%	108	14.2%	
Coexisting comorbidities					
Dysthymic disorder	304	10.0%	76	10.0%	0.0000
Peripheral vascular diseases	244	8.0%	61	8.0%	0.0000
Osteoporosis	760	25.0%	190	25.0%	−0.0667
Gout	561	18.4%	180	23.7%	−0.0522
Headache	1292	42.4%	323	42.4%	0.0000
Diabetic neuropathy	176	5.8%	49	6.4%	−0.0066
Rheumatoid arthritis	150	4.9%	51	6.7%	−0.0177
Pressure ulcer	82	2.7%	29	3.8%	−0.0112
Current smoking	72	2.4%	27	3.6%	−0.0118
ARD	143	4.7%	45	5.9%	−0.0122
CCI score					
Mean (SD)	0.79 ± 1.17		0.81 ± 1.20		0.0220
Median (Q1–Q3)	0.00 (0.00, 1.00)		0.00 (0.00, 1.00)		
CCI Score					−0.0028
0	1,812	59.5%	452	59.4%	
≧1	1,232	40.5%	309	40.6%	
CCI					
Congestive heart failure	226	7.4%	48	6.3%	0.0112
Dementia	99	3.3%	30	3.9%	−0.0069
Chronic pulmonary disease	550	18.1%	146	19.2%	−0.0112
Rheumatic disease	30	1.0%	14	1.8%	−0.0085
Liver disease	375	12.3%	99	13.0%	−0.0428
DM with complications	224	7.4%	56	7.4%	0.0000
Hemiplegia and paraplegia	0	00.0%	0	0.0%	0.0000
Renal disease	165	5.4%	44	5.8%	−0.0036
AIDS	0	0.0%	0	0.0%	0.0000
aDCSI score					
Mean (SD)	0.54 ± 1.24		0.60 ± 1.39		0.0460
Median (Q1–Q3)	0.00 (0.00, 0.00)		0.00 (0.00, 0.00)		
aDCSI score					0.0440
0	2,320	76.2%	577	75.8%	
1	303	10.0%	70	9.2%	
2	192	6.3%	49	6.4%	
≧3	229	7.5%	65	8.5%	
aDCSI					
Retinopathy	110	3.6%	38	5.0%	−0.0138
Nephropathy	223	7.3%	61	8.0%	−0.0069
Neuropathy	170	5.6%	43	5.7%	−0.0007
Cerebrovascular disease	149	4.9%	35	4.6%	0.0030
Cardiovascular disease	378	12.4%	108	14.2%	−0.0177
Peripheral vascular disease	117	3.8%	31	4.1%	−0.0023
Metabolic disease	20	0.7%	6	0.8%	−0.0013
Outcomes					
3-mo analgesic prescription					0.009
No	1,307	42.9%	287	37.7%	
Yes	1,737	57.1%	474	62.3%	
3-mo opioid prescription					0.014
No	3,021	99.2%	739	97.1%	
Yes	23	0.8%	22	2.9%	
6-mo analgesic prescription					0.013
No	2,243	73.7%	527	69.3%	
Yes	801	26.3%	234	30.8%	
6-mo opioid prescription					0.030
No	3,035	99.7%	756	99.3%	
Yes	9	0.3%	13	1.7%	

aDCSI, adapted Diabetes Complications Severity Index; AIDS, acquired immune deficiency syndrome; ARD, alcohol-related disease; ASA, American Society of Anesthesiology; CCI, Charlson Comorbidity Index; IQR, interquartile range; N, number; ORIF, open reduction internal fixation; SMD, standardized mean difference.

### 3.2. Analgesic prescription 3 months after surgery

To evaluate the rate of long-term analgesic use between sarcopenia and nonsarcopenia patients undergoing neuraxial anesthesia, we examined outpatient clinical records and postoperative analgesic usage for 6 months after surgery. The aORs and corresponding 95% CIs of 3-month analgesic and opioid use for the sarcopenia and nonsarcopenia groups undergoing elective surgery are presented in Tables 2 and 3. The rate of analgesic prescription was significantly higher in the sarcopenia group than in the nonsarcopenia group 3 months after surgery (aOR, 1.27; 95% CI, 1.05–1.52; Table 2). After adjusting for potential confounding factors, including age, sex, surgery type, medical center level, ASA-derived physical status, coexisting comorbidities, smoking status, ARD, CCI score, and aDCSI score, the aORs (95% CIs) of 3-month analgesic use for patients aged 51 to 70 years, those aged older than 70 years, those who underwent hip replacement, and those who underwent knee replacement were 2.07 (1.42–3.02), 2.04 (1.37–3.04), 7.12 (6.47–16.55), and 8.58 (5.82–16.77), respectively, when compared with patients aged younger than 30 years and those who underwent herniorrhaphy (Table 2).

**Table 2:** Logistic regression model of analgesic prescription 3 months after surgery under neuraxial anesthesia.

	3-mo analgesic prescription					
	Crude OR (95% CI)		*P*	Adjusted OR* (95% CI)		*P*
Sarcopenia						
Nonsarcopenia (ref.)						
Sarcopenia	1.24	(1.06, 1.46)	0.0091	1.27	(1.05, 1.53)	0.015
Age group, y (ref. < 30 y)						
30–50	1.37	(0.96, 1.96)	0.0551	1.61	(1.09, 2.37)	0.969
51–70	2.15	(1.55, 2.98)	<0.0001	2.07	(1.42, 3.02)	0.001
>70	2.21	(1.6, 3.07)	<0.0001	2.04	(1.37, 3.04)	0.005
Sex (ref. = female)						
Male	0.42	(0.37, 0.48)	<0.0001	1.04	(0.87, 1.24)	0.669
Surgery types (ref. = herniorrhaphy)						
Hip replacement	11.52	(7.85, 16.91)	<0.0001	7.12	(6.47, 16.55)	0.001
Knee replacement	20.18	(16.03, 25.41)	<0.0001	8.58	(5.82, 16.77)	<0.001
Lower-limb ORIF	6.52	(5.38, 7.89)	0.1031	7.40	(5.96, 9.2)	0.092
Lower-limb amputation	4.23	(2.65, 6.76)	0.0923	6.54	(3.68, 11.64)	0.944
Medical center (ref. = nonmedical center)						
Medical center	0.76	(0.66, 0.89)	0.000	0.84	(0.71, 1.01)	0.062
ASA physical status (ref. = 1)						
2	1.27	(1.02, 1.58)	0.5202	1.01	(0.76, 1.32)	0.279
3	1.80	(1.53, 2.12)	<0.0001	0.93	(0.73, 1.19)	0.744
4	1.38	(1.1, 1.71)	0.6634	1.14	(0.54, 1.22)	0.332
CCI (ref. = 0)	1.08	(0.95, 1.23)	0.2405	1.08	(0.9, 1.29)	0.412
≧1	1.08	(0.95, 1.23)	0.2405	1.08	(0.9, 1.29)	0.412
aDCSI (ref. = 0)						
1	1.28	(1.03, 1.6)	0.1024	1.01	(0.78, 1.33)	0.137
2	1.23	(0.94, 1.61)	0.321	0.76	(0.55, 1.05)	0.288
3	0.94	(0.74, 1.2)	0.1025	0.73	(0.51, 1.03)	0.170
Coexisting comorbidities						
Dysthymic disorder	1.24	(0.94, 1.63)	0.1315	0.92	(0.67, 1.27)	0.617
Peripheral vascular diseases	1.15	(0.85, 1.55)	0.3658	1.02	(0.71, 1.47)	0.901
Osteoporosis	1.84	(1.55, 2.18)	<0.0001	0.97	(0.79, 1.2)	0.785
Gout	1.57	(1.33, 1.86)	<0.0001	1.43	(0.77, 1.75)	0.221
Headache	1.54	(1.33, 1.78)	<0.0001	1.29	(0.69, 1.53)	0.314
Diabetic neuropathy	1.13	(0.86, 1.49)	0.3836	1.11	(0.78, 1.57)	0.566
Rheumatoid arthritis	1.88	(1.37, 2.57)	<0.0001	1.16	(0.81, 1.65)	0.429
Pressure ulcer	0.81	(0.56, 1.19)	0.2837	0.84	(0.54, 1.33)	0.463
Current smoking	1.21	(0.80, 1.83)	0.3565	1.58	(0.88, 2.54)	0.159
ARD	1.49	(1.09, 2.04)	0.0115	1.63	(0.84, 2.34)	0.128

*All covariates presented in Table 2 were adjusted.

aDCSI, adapted Diabetes Complications Severity Index; AIDS, acquired immune deficiency syndrome; ARD, alcohol-related disease; ASA, American Society of Anesthesiology; CCI, Charlson Comorbidity Index; CI, confidence interval; IQR, interquartile range; N, number; OR, odds ratio; ORIF, open reduction internal fixation; ref., reference group.

**Table 3:** Logistic regression model of opioid prescription 3 months after surgery under neuraxial anesthesia.

	3-mo opioid prescription					
	Crude OR (95% CI)		*P*	Adjusted OR* (95% CI)		*P*
Sarcopenia						
Nonsarcopenia (ref.)						
Sarcopenia	2.26	(1.05, 4.9)	0.0378	1.11	(1.05, 2.45)	0.012
Age group, y (ref. < 30 y)						
30–50	1.44	(0.44, 4.68)	0.045	1.03	(0.31, 3.47)	0.598
51–70	4.86	(1.75, 13.51)	0.0008	1.55	(0.5, 4.85)	0.247
>70	6.04	(2.17, 16.85)	<0.0001	1.34	(0.42, 4.31)	0.668
Sex (ref. = female)						
Male	0.40	(0.27, 0.59)	<0.0001	1.20	(0.76, 1.91)	0.441
Surgical types (ref. = herniorrhaphy)						
Hip replacement	1.03	(7.34, 507.4)	0.0636	6.21	(2.48, 11.89)	0.039
Knee replacement	11.50	(15.36, 809.26)	<0.0001	4.55	(3.68, 12.15)	<0.0001
Lower-limb ORIF	29.12	(4.01, 211.52)	0.9661	2.45	(0.41, 14.46)	0.073
Lower-limb amputation	11.68	(13.95, 894.09)	0.0002	2.73	(3.68, 291.23)	0.272
Medical center (ref. = nonmedical center)						
Medical center	2.07	(1.4, 3.08)	0.000	1.19	(0.46, 3.29)	0.381
ASA physical status (ref. = 1)						
2	2.50	(1.11, 5.64)	0.2039	1.53	(0.63, 3.74)	0.596
3	6.56	(3.66, 11.76)	<0.0001	2.38	(1.14, 4.98)	0.076
4	8.74	(4.37, 17.46)	<0.0001	2.64	(1.1, 6.35)	0.088
CCI (ref. = 0)						
≧1	2.67	(1.81, 3.95)	<0.0001	1.11	(0.7, 1.75)	0.662
aDCSI (ref. = 0)						
1	2.99	(1.53, 5.85)	0.8278	1.18	(0.58, 2.39)	0.674
2	5.08	(2.83, 9.09)	0.0109	2.00	(1.05, 3.79)	0.079
3	4.22	(2.36, 7.56)	0.0808	1.30	(0.58, 2.93)	0.954
Coexisting comorbidities						
Dysthymic disorder	2.53	(1.27, 5.03)	0.0082	1.20	(0.59, 2.43)	0.620
Peripheral vascular diseases	4.41	(2.34, 8.28)	<0.0001	1.77	(0.88, 3.57)	0.110
Osteoporosis	3.39	(2.19, 5.25)	<0.0001	1.31	(0.8, 2.13)	0.278
Gout	2.63	(1.7, 4.07)	<0.0001	1.30	(0.82, 2.07)	0.268
Headache	2.89	(1.94, 4.3)	<0.0001	1.66	(0.88, 2.54)	0.220
Diabetic neuropathy	3.53	(1.88, 6.63)	<0.0001	1.08	(0.52, 2.25)	0.842
Rheumatoid arthritis	4.75	(2.59, 8.72)	<0.0001	2.08	(0.81, 3.9)	0.123
Pressure ulcer	3.74	(1.81, 7.73)	0.0004	1.51	(0.63, 3.6)	0.352
Current smoking	0.87	(0.28, 2.76)	0.8174	1.24	(0.38, 4.06)	0.721
ARD	1.35	(0.65, 2.78)	0.4194	1.45	(0.67, 3.13)	0.349

* All covariates presented in Table 2 were adjusted.

aDCSI, adapted Diabetes Complications Severity Index; AIDS, acquired immune deficiency syndrome; ARD, alcohol-related disease; ASA, American Society of Anesthesiology; CCI, Charlson Comorbidity Index; CI, confidence interval; IQR, interquartile range; N, number; OR, odds ratio; ORIF, open reduction internal fixation; ref., reference group.

### 3.3. Opioid prescription 3 months after surgery

The rate of opioid prescriptions was notably greater in the sarcopenia group compared with the nonsarcopenia group (Table 1) at the 3-month follow-up after surgery, with an aOR of 1.11 (95% CI, 1.05–2.45), as presented in Table 3. After adjustment, the aORs (95% CI) of 3-month opioid use for the patients who underwent hip replacement and those who underwent knee replacement were 6.21 (2.48–11.89) and 4.55 (3.68–12.15) compared with those who underwent herniorrhaphy (Table 3).

### 3.4. Analgesic prescription 6 months after surgery

After the 6-month follow-up, the risk of receiving analgesic prescriptions was found to be significantly greater in the sarcopenia group compared with the nonsarcopenia group (aOR, 1.17; 95% CI, 1.07–1.42; Table 4). After adjustment for the aforementioned cofounding factors, the aORs (95% CI) of 6-month analgesic use in the patients who underwent hip replacement and those who underwent knee replacement were 3.83 (2.57–5.71) and 5.66 (4.32–7.41), respectively, compared with those who underwent herniorrhaphy (Table 4).

**Table 4:** Logistic regression model of analgesic prescription 6 months after surgery under neuraxial anesthesia.

	6-mo analgesic prescription					
	Crude OR (95% CI)		*P*	Adjusted OR* (95% CI)		*P*
Sarcopenia						
Nonsarcopenia (ref.)						
Sarcopenia	1.24	(1.04, 1.48)	0.014	1.17	(1.07, 1.42)	0.019
Age group, years (ref. < 30 y)						
30–50	1.68	(0.97, 2.93)	0.193	1.45	(0.82, 2.56)	0.678
51–70	3.68	(2.2, 6.14)	<0.0001	2.00	(1.16, 3.45)	0.003
>70	3.73	(2.23, 6.22)	<0.0001	1.85	(1.06, 3.23)	0.048
Sex (ref. = female)						
Male	0.55	(0.47, 0.63)	<0.0001	1.05	(0.87, 1.26)	0.634
Surgical types (ref. = herniorrhaphy)						
Hip replacement	3.77	(2.58, 5.53)	0.0006	3.83	(2.57, 5.71)	0.001
Knee replacement	6.57	(5.18, 8.32)	<0.0001	5.66	(4.32, 7.41)	<0.0001
Lower-limb ORIF	2.36	(1.87, 2.98)	0.5809	2.47	(1.91, 3.19)	0.379
Lower-limb amputation	0.97	(0.47, 1.99)	0.303	1.10	(0.49, 2.47)	0.225
Medical center (ref. = nonmedical center)						
Medical center	0.81	(0.68, 0.96)	0.015	0.84	(0.7, 1.02)	0.075
ASA physical status (ref. = 1)						
2	1.81	(1.39, 2.36)	0.5946	1.28	(0.95, 1.73)	0.446
3	2.37	(1.93, 2.91)	<0.0001	1.23	(0.94, 1.6)	0.739
4	2.12	(1.63, 2.76)	0.0137	1.32	(0.94, 1.84)	0.343
CCI (ref = 0)						
≧1	1.30	(1.12, 1.5)	0.0004	1.16	(0.97, 1.38)	0.096
aDCSI (ref. = 0)						
1	1.64	(1.31, 2.05)	0.0006	1.34	(0.94, 1.72)	0.233
2	1.42	(1.07, 1.88)	0.1056	0.93	(0.67, 1.28)	0.679
3	0.84	(0.63, 1.12)	0.0028	0.74	(0.51, 1.05)	0.133
Coexisting comorbidities						
Dysthymic disorder	1.63	(1.24, 2.16)	0.0006	1.21	(0.89, 1.63)	0.223
Peripheral vascular diseases	1.24	(0.91, 1.7)	0.1802	1.18	(0.83, 1.69)	0.362
Osteoporosis	1.83	(1.54, 2.17)	<0.0001	1.11	(0.91, 1.35)	0.295
Gout	1.79	(1.51, 2.12)	<0.0001	1.14	(0.176, 1.7)	0.348
Headache	1.74	(1.49, 2.02)	<0.0001	1.36	(0.75, 1.60)	0.280
Diabetic neuropathy	0.93	(0.68, 1.26)	0.621	0.84	(0.59, 1.22)	0.363
Rheumatoid arthritis	2.38	(1.79, 3.18)	<0.0001	1.21	(0.74, 2.11)	0.325
Pressure ulcer	0.73	(0.46, 1.16)	0.1819	0.91	(0.54, 1.53)	0.720
Current smoking	1.00	(0.64, 1.57)	0.987	1.33	(0.82, 2.15)	0.251
ARD	0.76	(0.54, 1.08)	0.1257	0.82	(0.56, 1.2)	0.297

* All covariates presented in Table 2 were adjusted.

aDCSI, adapted Diabetes Complications Severity Index; AIDS, acquired immune deficiency syndrome; ARD, alcohol-related diseases; ASA, American Society of Anesthesiology; CCI, Charlson Comorbidity Index; CI, confidence interval; IQR, interquartile range; N, number; OR, odds ratio; ORIF, open reduction internal fixation; ref., reference group.

### 3.5. Opioid prescription 6 months after surgery

After 6 months postsurgery, the sarcopenia group showed a significantly higher rate of opioid prescription compared with the nonsarcopenia group (aOR, 1.89; 95% CI, 1.12–4.96; Table 5). After adjustment for the aforementioned covariates listed in Table 2, the aOR (95% CI) of 6-month opioid use for the patients who underwent hip replacement was 7.95 (4.25–9.82) compared with those who underwent herniorrhaphy (Table 5). We have incorporated sample sizes (N) into Tables 2–5, which are presented as Supplemental Table 1, http://links.lww.com/PR9/A217.

**Table 5:** Logistic regression model of opioid prescription 6 months after surgery under neuraxial anesthesia.

	6-mo opioid prescription					
	Crude OR (95% CI)		*P*	Adjusted OR* (95% CI)		*P*
Sarcopenia						
Nonsarcopenia (ref.)						
Sarcopenia	3.80	(1.5, 9.65)	0.005	1.89	(1.12, 4.96)	0.019
Age group, years (ref. < 30 y)						
30–50	2.56	(0.29, 22.92)	0.3401	1.62	(0.17, 15.1)	0.740
51–70	9.05	(1.22, 67.28)	0.0122	3.31	(0.39, 27.84)	0.116
>70	10.65	(1.43, 79.6)	0.003	2.40	(0.27, 21.15)	0.539
Sex (ref. = female)						
Male	0.61	(0.34, 1.09)	0.0947	1.58	(0.78, 3.18)	0.201
Surgical types (ref. = herniorrhaphy)						
Hip replacement	20.35	(5.08, 81.53)	0.0033	7.95	(4.25, 9.82)	0.001
Knee replacement	12.56	(3.63, 43.4)	0.0292	7.70	(2.05, 28.86)	0.137
Lower-limb ORIF	4.15	(1.22, 14.1)	0.0644	5.00	(1.41, 17.72)	0.850
Lower-limb amputation	13.84	(2.79, 68.73)	0.1475	3.45	(0.51, 23.35)	0.623
Medical center (ref. = nonmedical center)						
Medical center	1.79	(0.98, 3.25)	0.057	1.67	(0.9, 3.12)	0.104
ASA physical status (ref. = 1)						
2	1.75	(0.44, 6.99)	0.1414	0.82	(0.18, 3.65)	0.185
3	6.57	(2.69, 16.02)	0.0072	2.10	(0.69, 6.33)	0.223
4	11.76	(4.34, 31.84)	<0.0001	2.10	(0.88, 6.96)	0.135
CCI (ref. = 0)						
≧1	3.40	(1.89, 6.12)	<0.0001	1.58	(0.79, 3.16)	0.196
aDCSI (ref. = 0)						
1	4.03	(1.67, 9.76)	0.2951	1.72	(0.68, 4.36)	0.560
2	3.23	(1.13, 9.23)	0.7236	1.29	(0.43, 3.89)	0.856
3	4.73	(2.06, 10.85)	0.1135	1.68	(0.56, 5.06)	0.640
Coexisting comorbidities						
Dysthymic disorder	6.48	(3.11, 13.49)	<0.0001	3.46	(0.61, 7.51)	0.442
Peripheral vascular diseases	3.49	(1.25, 9.77)	0.0174	1.35	(0.44, 4.14)	0.600
Osteoporosis	3.57	(1.87, 6.82)	0.0001	1.70	(0.82, 3.56)	0.157
Gout	3.09	(1.64, 5.81)	0.0005	1.48	(0.75, 2.91)	0.261
Headache	1.71	(0.9, 3.26)	0.1032	0.80	(0.4, 1.6)	0.530
Diabetic neuropathy	2.05	(0.63, 6.61)	0.2315	0.51	(0.14, 1.84)	0.300
Rheumatoid arthritis	3.40	(1.22, 9.52)	0.0198	1.40	(0.48, 4.07)	0.537
Pressure ulcer	4.21	(1.5, 11.81)	0.0062	2.10	(0.61, 7.22)	0.238
Current smoking	1.33	(0.32, 5.49)	0.6942	1.53	(0.35, 6.61)	0.573
ARD	1.53	(0.55, 4.27)	0.4202	1.10	(0.36, 3.34)	0.866

*All covariates presented in Table 2 were adjusted.

aDCSI, adapted Diabetes Complications Severity Index; AIDS, acquired immune deficiency syndrome; ARD, alcohol-related disease; ASA, American Society of Anesthesiology; CCI, Charlson Comorbidity Index; CI, confidence interval; IQR, interquartile range; N, number; OR, odds ratio; ORIF, open reduction internal fixation; ref., reference group.

## 4. Discussion

Numerous challenges and severe surgical complications are experienced by many patients with sarcopenia.^14^ However, it remains uncertain whether preoperative sarcopenia is associated with CPSP and long-term analgesic use. This is, to our knowledge, the first and largest study that used a PSM method to compare the long-term postoperative analgesic use of patients with and without sarcopenia. This retrospective cohort study, based on real-world data, included 3805 surgical patients, with 761 diagnosed with sarcopenia and 3044 without. The analysis revealed significant differences in postoperative analgesic and opioid prescription rates between these 2 groups. Notably, at 3 months postsurgery, 62.3% of patients with sarcopenia received analgesics (2.9% opioids), whereas 57.1% of those without sarcopenia received analgesics (0.8% opioids). Similarly, at 6 months postsurgery, 30.8% of sarcopenic patients received analgesics (1.7% opioids), compared with 26.3% of nonsarcopenic patients receiving analgesics (0.3% opioids). Multivariable logistic regression analyses demonstrated that patients with sarcopenia undergoing neuraxial anesthesia surgery were significantly more likely to receive analgesic prescriptions at both 3 months (aOR 1.27; 95% CI 1.05–1.53) and 6 months (aOR 1.17; 95% CI 1.07–1.42) postoperatively, compared with those without sarcopenia. Moreover, sarcopenic patients exhibited significantly higher opioid prescription rates at 3 months (aOR 1.11; 95% CI 1.05–2.45) and 6 months (aOR 1.89; 95% CI 1.12–4.96) postsurgery. In addition, advanced age (above 50 years) and specific surgical procedures, such as hip and knee replacements, were associated with a higher likelihood of persistent analgesic and opioid consumption. These findings offer insights into postoperative pain management in the context of sarcopenia, emphasizing the importance of addressing chronic postsurgical pain and reducing long-term analgesic or opioid use, particularly among sarcopenic patients. Recognizing the impact of age and specific surgical procedures on postoperative pain management is vital for optimizing patient care and outcomes. This study adds valuable information to the existing literature, guiding future research and clinical practices.^16,20,24^

Regarding frailty, we acknowledge its significant impact on postoperative outcomes, including pain experiences and analgesic usage. Regrettably, owing to the limitations of our data source, we were unable to directly conduct a comprehensive analysis of frailty. Nonetheless, we endeavored to mitigate this limitation by considering relevant covariates that might indirectly encompass aspects of frailty in our study. Specifically, we incorporated ASA scores and assessed various coexisting comorbidities, including conditions such as dysthymic disorder, peripheral vascular diseases, osteoporosis, gout, headache, diabetic neuropathy, rheumatoid arthritis, pressure ulcer, and alcohol-related diseases, along with the Charlson Comorbidity Index (CCI) score. These variables reflect patients' physical status and the presence of comorbid conditions, which can indirectly signify frailty.^23,29^ This approach facilitated the matching and control for potential confounding factors within our PSM analysis. While we could not directly measure frailty, these surrogate measures helped us indirectly address its influence in our study.

Analyzing the patterns of analgesic consumption at both the 3-month and 6-month postsurgery intervals is a critical component of our research. Since analgesic usage is used as a surrogate measure for assessing CPSP, a thorough examination of the variations in analgesic consumption at 3 months between sarcopenia and nonsarcopenia patients, while informative, may not provide a complete picture.^15^ Therefore, a more rigorous approach entails investigating the distinctions in analgesic consumption at the 6-month postsurgery point. This scrutiny reinforces the presence of CPSP, particularly within the context of sarcopenic and nonsarcopenic patients. Our study consistently demonstrates that regardless of whether we evaluate analgesic consumption at 3 months or 6 months, a notably higher proportion of CPSP cases are observed among sarcopenic patients. This trend is particularly prominent within the subgroup of patients who were prescribed opioids. Importantly, it is essential to highlight that patients in our cohort did not use opioids before the surgery; opioid use only commenced postoperatively. The strict regulation and limited accessibility of opioids, which are typically not available from standard pharmacies, render their postoperative usage a robust indicator. Consequently, when we analyze opioid usage at both the 3-month and 6-month marks, we can confidently assert that sarcopenic patients face a significantly increased risk of experiencing CPSP. Focusing on opioids at both time points enhances the robustness of CPSP as the primary outcome in our study.

Sarcopenia, characterized by the age-related loss of muscle mass and function, is a complex condition influenced by a range of factors, including the natural process of aging, physical inactivity, inadequate nutrition, hormonal shifts, chronic illnesses, neurological disorders, medications, and genetic predispositions.^7,8,21^ It is essential for both researchers and clinicians to acknowledge and address these multifaceted causes when diagnosing and managing sarcopenia. Moreover, the presence of confounding factors, such as comorbid medical conditions, medication use, nutritional status, physical activity levels, hormonal changes, body composition, and social determinants of health, can significantly affect the relationship between sarcopenia and health outcomes.^7–9,21^ In our study, we made a comprehensive effort to account for these potential confounding factors by using PSM. This approach allowed us to match patients based on a wide range of variables, including age, sex, type of surgery, the level of the medical center, ASA-derived physical status, and various coexisting comorbidities (such as dysthymic disorder, peripheral vascular diseases, osteoporosis, gout, headache, diabetic neuropathy, rheumatoid arthritis, pressure ulcer, and alcohol-related diseases). In addition, we considered smoking status, CCI score, and aDCSI score. These covariates encompass factors available in our NHIRD to minimize potential biases and enhance the accuracy of our results. However, it is crucial to acknowledge that certain factors, such as genetic predispositions, are beyond the scope of our data source. This limitation is transparently included in our study. While the causes of sarcopenia are indeed diverse, our research primarily concentrated on the impact of postoperative analgesic consumption, rather than providing an exhaustive exploration of the underlying causes of sarcopenia.

Currently, the causal relationship between sarcopenia and CPSP remains unclear, as does the relevant mechanism of action. Our results indicate a higher probability of developing CPSP in patients with preoperative sarcopenia due to increased long-term analgesic use. Sarcopenia is a multifaceted syndrome with a complex pathogenesis.^14^ CPSP, a special type of chronic pain, also has a complex and unclear pathogenesis similar to sarcopenia, and the related research is still in the exploratory stage.^15,27^ Sarcopenia and CPSP share common mechanisms, such as an imbalance between the inflammatory response and oxidative stress.^3^ In addition, the ubiquitinated proteasome system plays a crucial role in muscle fiber degradation, and its overactivation can lead not only to sarcopenia but also to chronic pain.^22^ The same causative gene may also be responsible for both conditions. A UK Biobank cohort study reported that homozygous *HFEC282Y* was strongly associated with sarcopenia and chronic pain in older patients.^26^ Therefore, patients with sarcopenia might develop CPSP and thus require the long-term use of analgesics, especially opioids.

While there is currently a dearth of research exploring the potential correlation between sarcopenia and prolonged analgesic and opioid use, a plethora of studies have established a noteworthy relationship between sarcopenia and increased occurrence of unfavorable postoperative outcomes when compared with individuals who do not have sarcopenia.^9^ Our findings demonstrated that sarcopenia is an independent risk factor for 30-day and 90-day adverse postoperative outcomes, such as postoperative pneumonia, bleeding, septicemia, and mortality. Postoperative complications tend to prolong the duration of postoperative pain, leading to the prolonged use of analgesics after surgery.^11^ Therefore, this may be a reason for long-term analgesic use in patients with sarcopenia.

The number of studies on postoperative pain in older patients with sarcopenia is limited. Most of them have focused on acute postoperative pain, and whether sarcopenia affects acute postoperative pain in older patients remains inconclusive.^18^ Generally, older patients are less sensitive to pain than younger patients.^28^ However, in this study, we found a significant increase in analgesic and opioid use in the older patients with sarcopenia. After adjustment for age, sex, surgery type, medical center level, ASA-derived physical status, coexisting comorbidities, smoking, ARD, CCI scores, and aDCSI scores, this conclusion still remains valid, signifying that advanced age represents an autonomous hazard element for extended postoperative utilization of analgesics among patients. Although the reason for this phenomenon is not clear, the results suggest that older patients who undergo neuraxial anesthesia are more likely to have long-term postoperative pain. Therefore, CPSP in older patients with sarcopenia warrants special attention.

In this study, we determined that the patients undergoing different types of surgery under neuraxial anesthesia had different rates of long-term analgesic and opioid use. The patients who underwent knee and hip replacement had significantly higher rates of long-term analgesic and opioid use than did those who underwent herniorrhaphy, indicating that these patients experience postoperative pain for a longer period. The incidence of chronic pain was 7% to 34% in patients undergoing lower-extremity joint replacement surgery^31^; this finding is compatible with the higher rates of long-term analgesic and opioid use observed in the patients undergoing these types of surgeries than in those undergoing herniorrhaphy. Notwithstanding, the timeframe during which sarcopenia exerts an amplified impact on chronic postsurgical pain (CPSP) after hip and knee replacement surgeries is yet to be established, as the investigation discovered no discernible divergence in pain scores based on the Western Ontario and McMaster Universities Osteoarthritis Index 10 months after knee replacement surgery between those with and without sarcopenia.^18^

The long-term use of analgesics can cause many side effects, such as stomach bleeding, liver damage, and kidney damage.^16,20^ The long-term use of opioids is associated with tolerance, addiction, and even cancer progression, which can contribute to the development of cancer.^24^ The aforementioned side effects might be observed in patients with long-term analgesic and opioid use (those with sarcopenia, older patients, and those undergoing hip and knee replacement) according to our findings (Tables 2–5). To prevent these side effects, reasonable and effective perioperative analgesic management or improved surgical procedures, such as microinvasive surgeries, that result in a minor wound with less pain might be beneficial for patients with sarcopenia, especially older patients and those undergoing hip and knee replacement.^27^ These surgical or anesthetic techniques, including multimodal analgesia, minimally invasive surgery, and nerve block, might be associated with a reduction in acute postsurgical pain that contributes to CPSP.^27^

This study used data from the NHIRD, which reliably records the detailed medical information of NHI enrollees, and this database has been used in many high-quality studies.^24,25,32^ The present comparative study used a large PSM-based design to maintain balance among confounders of case and control groups, resulting in the absence of bias (Table 1). This study presents several noteworthy limitations that require careful consideration. First, while PSM was thoughtfully used to enhance the comparability of study groups, it remains susceptible to the influence of unmeasured confounding factors. Second, the study's exclusive inclusion of patients of Asian descent raises questions about the generalizability of its findings to populations of different ethnic backgrounds. However, it is important to note that previous reports have not shown significant variations in the occurrence of CPSP between Asian and non-Asian populations. Nonetheless, exercising caution is advisable when extending these findings beyond Asian cohorts. Third, the study's use of analgesic consumption as an indicator for Chronic Postsurgical Pain (CPSP) is influenced by limitations inherent in the Numeric Rating Scale (NRS). The NRS is prone to recall bias and is less effective in patients with lower educational backgrounds, as well as those facing communication challenges due to nonverbal or cognitive impairments. Moreover, it may not adequately capture the nuances of complex or chronic pain conditions.^12,17^ To address these constraints, the study chose analgesic consumption as a surrogate measure for CPSP, with the understanding that it may not encompass instances where patients endure pain without seeking medication. Nonetheless, it is essential to highlight that the study's meticulous inclusion and exclusion criteria are anticipated to mitigate the potential impact of this approach on the study's findings. Fourth, an acknowledged limitation is the study's inability to definitively establish a direct correlation between pain experienced at 3 or 6 months postsurgery and its direct relation to the surgical site. While concerns about potential complications and new hospitalizations, as raised by the reviewer, were not observed in the patient cohort, as the study explicitly excluded readmissions related to postoperative complications, it is important to recognize that using analgesic consumption as a surrogate parameter for CPSP represents an indirect approach and does not offer a direct assessment of pain. These limitations emphasize the need for caution when drawing definitive conclusions regarding the relationship between sarcopenia and CPSP based solely on analgesic consumption. Fifth, the lack of detailed information on certain variables such as lifestyle factors, nutrition, dietary data, laboratory results, and genetic predispositions within the NHIRD limits our ability to fully account for all potential confounding factors. Nevertheless, it is worth noting that previous studies have reported the accuracy and quality of data from NHIRD, which provides a level of confidence in the database's reliability.^4,5,13^ Finally, it is important to note that the research is limited to patients who underwent neuraxial anesthesia, and the findings may not be universally applicable to individuals receiving alternative anesthesia modalities.

## 5. Conclusion

Our study highlights sarcopenia as a distinctive risk factor for prolonged analgesic use after surgery with neuraxial anesthesia. Patients diagnosed with sarcopenia face an elevated risk of extended reliance on analgesics and opioids. These findings emphasize the clinical importance of identifying and addressing sarcopenia in surgical patients undergoing neuraxial anesthesia procedures, offering potential avenues to optimize postoperative pain management and improve patient outcomes.

## Disclosures

The authors have no conflict of interest to declare.

## Supplementary Material

SUPPLEMENTARY MATERIAL
