# Supplementary material for: Persistence of analgesic usage and opioid consumption in sarcopenic patients undergoing neuraxial anesthesia: a nationwide retrospective cohort study
Source: Pain Rep. 2024 Mar 8;9(2):e1129. doi: 10.1097/PR9.0000000000001129 (PMC10927332; doi:10.1097/PR9.0000000000001129)
Supplement: SUPPLEMENTARY MATERIAL [file painreports-9-e1129-s001.pdf]

Supplemental Table 1. Analgesic and Opioid Prescription Rates at 3 and 6 Months Following Neuraxial Anesthesia Surgery

	Nonsarcopenia		Sarcopenia		*P* Value
	N = 3044		N = 761		
	N	%	N	%	
**3-month analgesic prescription**					0.009
No	1,307	42.9%	287	37.7%	
Yes	1,737	57.1%	474	62.3%	
**3-month opioid prescription**					0.014
No	3,021	99.2%	739	97.1%	
Yes	23	0.8%	22	2.9%	
**6-month analgesic prescription**					0.013
No	2,243	73.7%	527	69.3%	
Yes	801	26.3%	234	30.8%	
**6-month opioid prescription**					0.030
No	3,035	99.7%	756	99.3%	
Yes	9	0.3%	13	1.7%	

Abbreviations: N, number

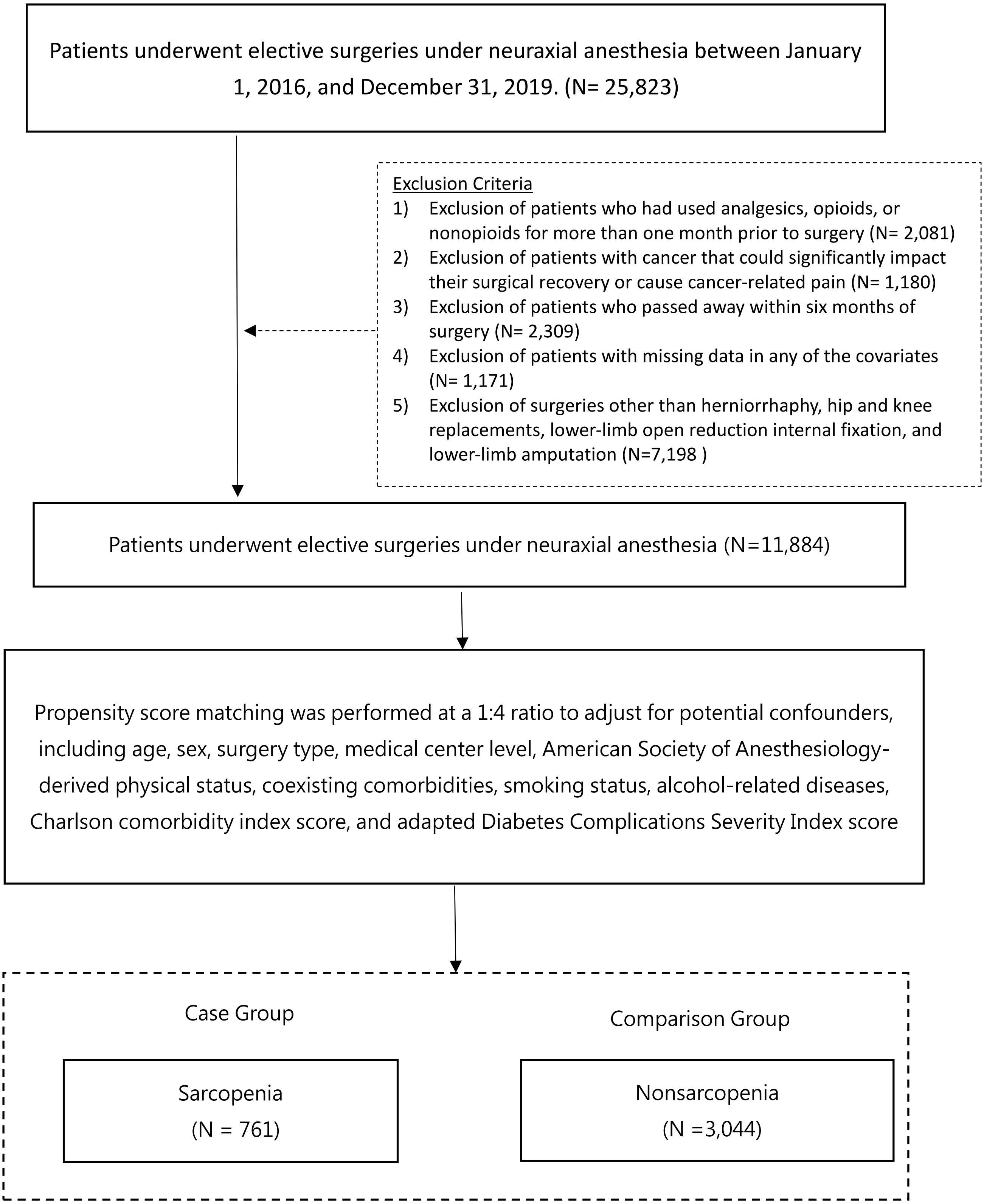
Patients underwent elective surgeries under neuraxial anesthesia between January 1, 2016, and December 31, 2019. (N= 25,823)
Exclusion Criteria
1) Exclusion of patients who had used analgesics, opioids, or nonopioids for more than one month prior to surgery (N= 2,081)
2) Exclusion of patients with cancer that could significantly impact their surgical recovery or cause cancer-related pain (N= 1,180)
3) Exclusion of patients who passed away within six months of surgery (N= 2,309)
4) Exclusion of patients with missing data in any of the covariates (N= 1,171)
5) Exclusion of surgeries other than herniorrhaphy, hip and knee replacements, lower-limb open reduction internal fixation, and lower-limb amputation (N=7,198)
Patients underwent elective surgeries under neuraxial anesthesia (N=11,884)
Propensity score matching was performed at a 1:4 ratio to adjust for potential confounders, including age, sex, surgery type, medical center level, American Society of Anesthesiology-derived physical status, coexisting comorbidities, smoking status, alcohol-related diseases, Charlson comorbidity index score, and adapted Diabetes Complications Severity Index score
Case Group
Comparison Group
Sarcopenia
(N = 761)
Nonsarcopenia
(N =3,044)
